# Intracellular Function of Interleukin-1 Receptor Antagonist in Ischemic Cardiomyocytes

**DOI:** 10.1371/journal.pone.0053265

**Published:** 2013-01-08

**Authors:** Elena Vecile, Aldo Dobrina, Fadi N. Salloum, Benjamin W. Van Tassell, Antonella Falcione, Edoardo Gustini, Samuele Secchiero, Sergio Crovella, Gianfranco Sinagra, Nicoletta Finato, Martin J. Nicklin, Antonio Abbate

**Affiliations:** 1 Department of Life Sciences, University of Trieste, Italy; 2 Victoria Johnson Research Laboratory and VCU Pauley Heart Center, Virginia Commonwealth University, Richmond, Virginia, United States of America; 3 Institute for Maternal and Child Health-IRCCS Burlo Garofolo, Trieste, Italy; 4 Cardiovascular Department, University of Trieste, Italy; 5 Department of Medical and Morphological Research, University of Udine, Italy; 6 Division of Genomic Medicine, Sir Henry Wellcome Laboratories for Medical Research, University of Sheffield, United Kingdom; University of Torino, Italy

## Abstract

**Background:**

Loss of cardiac myocytes due to apoptosis is a relevant feature of ischemic heart disease. It has been described in infarct and peri-infarct regions of the myocardium in coronary syndromes and in ischemia-linked heart remodeling. Previous studies have provided protection against ischemia-induced cardiomyocyte apoptosis by the anti-inflammatory cytokine interleukin-1 receptor-antagonist (IL-1Ra). Mitochondria triggering of caspases plays a central role in ischemia-induced apoptosis. We examined the production of IL-1Ra in the ischemic heart and, based on dual intra/extracellular function of some other interleukins, we hypothesized that IL-1Ra may also directly inhibit mitochondria-activated caspases and cardiomyocyte apoptosis.

**Methodology/Principal Findings:**

Synthesis of IL-1Ra was evidenced in the hearts explanted from patients with ischemic heart disease. In the mouse ischemic heart and in a mouse cardiomyocyte cell line exposed to long-lasting hypoxia, IL-1Ra bound and inhibited mitochondria-activated caspases, whereas inhibition of caspase activation was not observed in the heart of mice lacking IL-1Ra (Il-1ra−/−) or in siRNA to IL-1Ra-interfered cells. An impressive 6-fold increase of hypoxia-induced apoptosis was observed in cells lacking IL-1Ra. IL-1Ra down-regulated cells were not protected against caspase activation and apoptosis by knocking down of the IL-1 receptor, confirming the intracellular, receptor-independent, anti-apoptotic function of IL-1Ra. Notably, the inhibitory effect of IL-1Ra was not influenced by enduring ischemic conditions in which previously described physiologic inhibitors of apoptosis are neutralized.

**Conclusions/Significance:**

These observations point to intracellular IL-1Ra as a critical mechanism of the cell self-protection against ischemia-induced apoptosis and suggest that this cytokine plays an important role in the remodeling of heart by promoting survival of cardiomyocytes in the ischemic regions.

## Introduction

Interleukin-1 (IL-1) receptor antagonist inhibits the inflammatory effects of IL-1α and IL-1β by competing for IL-1 type-I membrane receptor (IL-1R1) [1], [2]. Recently, an often lethal autoinflammatory syndrome in children (DIRA) [3] has been linked to genetic deficiency of IL-1Ra. Besides a secreted protein, three intracellular, unsecreted isoforms of IL-1Ra have been described in humans, and in mouse tissues both a secreted and an intracellular isoform have been confirmed [4]. Whereas extracellular IL-1Ra inhibits IL-1 activity by binding to IL-1R1, intracellular IL-1Ra was recently evidenced to inhibit phosphorilation of proteins involved in IL-1R1 signal transduction in keratinocytes [5]. Increased serum levels of IL-1Ra have been found to precede the appearance of markers of heart necrosis and of inflammation in patients with myocardial ischemic disease [6], [7], suggesting that cardiac myocytes in ischemic heart regions may synthesize cytokines which influence cell survival. Ischemia-induced apoptosis is a relevant feature in ischemic heart disease [8]–[10]. Previous studies have provided cardioprotection by IL-1Ra against ischemia-induced cardiomyocyte apoptosis, which was primarily based on the anti-inflammatory, extracellular function of IL-1Ra, either by inducing overexpression of IL-1Ra [11] or by administration of recombinant IL-1Ra [12]. Moreover, in recent studies substantial cardioprotection against the ischemic damage was evidenced in coronary ligation experiments performed on mice lacking the IL-1R1 [13], not responsive to IL-1. Other members of IL-1 family, IL-1α [14] and IL-33 [15], are nuclear proteins that are released into the extracellular space. This observation led to define these cytokines as dual-function, intra/extracellular molecules [16]. Goal of the study was to examine the production of IL-1Ra by cardiac myocytes in ischemic heart disease and to investigate whether endogenous IL-1Ra may influence cell apoptosis by additional mechanisms besides IL-1Ra recognized anti-IL-1 function at the IL-1R1 level.

## Methods

### Patients

Human samples were collected after written informed consent was obtained in accordance with the Declaration of Helsinki and with approval by the Independent Ethics Committee of the University of Udine, Udine, Italy. Myocardial samples were taken from explanted hearts in 5 patients with ischemic cardiomyopathy and prior AMI undergoing heart transplantation. All patients had end-stage heart failure (NYHA class IV) and severely impaired systolic function (left ventricular ejection fraction <20%), and had been on a waiting list for transplantation for more than 12 months. Samples were taken from the explanted hearts in the areas adjacent to old post infarct scars, in intermediate regions, and in remote regions. The peri-infarct scar area was defined as the zone bordering the infarct scar in the left ventricle where viable myocardium was prevalent and reparative fibrosis only marginal. Intermediate was defined the area 1 cm distant from the scar, and remote regions were areas with macroscopic features of normal blood supply and trophism, several cm distant from infarct scars but within the same heart ventricle. Samples were frozen at –80°C within 30 minutes after heart explant, and subsequently analyzed. Hearts were also taken from a control group of four subjects who died as consequence of head trauma, and were virtually free of cardiac disease. In these subjects, hearts were taken at autopsy shortly after death and heart samples set up for detection of apoptosis.

### Coronary Ligation Model

Procedures were approved by the Animal Care and Use Committee of Virginia Commonwealth University using US National Institutes of Health (NIH) guidelines (No. 85-23, revised 1996). C57BL/6 male 8-week-old mice (Harlan Sprague Dawley, Indianapolis, IN) anesthetized with 50–70 mg per kg body weight pentobarbital were intubated and subjected to ligation of the proximal left coronary artery, as previously described [12]. Additional animals underwent a sham operation including every step except coronary ligation. After completion of the infarction protocol, animals were sacrificed and hearts were excised and stored at −80°C.

### Il-1ra −/− Model

Procedures were approved by the institutional Animal Research Committee of the University of Trieste, using NIH guidelines. Mutant mice lacking IL-1Ra (Il-1ra−/−) were generated as described previously [17]. All comparisons were made to littermate controls of identical genetic background (C57BL/6J). Post mortem heart changes were evaluated on male 8-week-old WT (Il-1ra+/+) or Il-1ra−/− mouse hearts, which were washed by perfusion with PBS and rapidly excised from animals as previously described [12]. Briefly, after sacrifice the abdominal aorta was cannulated with a polyethylene catheter and filled with PBS and the right atrium was cut to allow drainage. Hearts were then excised from animals and immersed in 1–2 drops of PBS on bottom of polyethylene tubes, and were then incubated at 37°C in hypoxic (95%N_2_–5%CO_2_) conditions in a Micro galaxy, RS (Biotech) incubator for various periods of time. At each time-point, hearts were harvested at −80°C. Control samples underwent the same treatment except incubation.

### Cell Culture

Experiments were performed on HL-1 cells, a mouse cardiac muscle cell line that retains phenotypic characteristics of adult cardiomyocytes, gift of Dr. W.C. Claycomb [18]. Culture conditions and media were as previously described [19]. Cells were grown to confluence on 25 cm^2^ flasks for enzyme assays, or on 22-mm glass coverslips placed on the bottom of 35-mm Petri dishes for immunofluorescence studies. Cells were then incubated at 37°C either in normoxia or in hypoxia (95% N_2_-5% CO_2_) conditions for up to 9 hr. Cells were then washed and immediately frozen at −80°C (flasks), or −20°C (coverslips).

### siRNA Transfection

HL-1 cells were transfected with siRNA targeted against IL-1Ra and/or IL-1R1 mRNA. siRNA duplexes targeted against IL-1Ra (sc-39618-A,-B,-C) or IL-1R1 (sc-35652), or control (sc-37007, sc-44233) mRNA, and transfection reagents and media were obtained from Santa Cruz Biotechnology Inc., CA. Cells grown on 6-well tissue culture plates or glass coverslips were treated for 18 hr with 1 ug targeted siRNA, according to the manufacturer’s transfection protocol. At 18 h post transfection, cell dishes or coverslips were incubated at 37°C either in normoxia or in hypoxia (95% N_2_ 5% CO_2_) conditions, and then harvested as described above. Transfection efficiency was evaluated by immunostaining of glass coverslips with anti-IL-1Ra and/or anti-IL-1R1 monoclonal Abs and by Western blot analysis for IL-1Ra and IL-1R1 protein in transfected cell lysates, and compared to untreated and control siRNA-treated cells. In addition, functional inactivation of the IL-1R1 was assayed by RTqPCR analysis of IL-6 RNA expression [5] in transfected cells after incubation of the cells in the presence or absence of IL-1β, and compared to untreated or mismatch siRNA (control) treated cells.

### Immunohistochemistry and Immunofluorescence

Cryostat sections of myocardial tissue were fixed for 5 min in acetone and then incubated for 2 hr in PBS 20% FCS. Unless specified, Abs were purchased from Santa Cruz. For immunofluorescence studies, treatment with goat anti-C20 human IL-1Ra Ab was followed by donkey FITC-conjugated anti-goat Ab. Endothelial cells were stained by mouse monoclonal anti-CD31/PECAM-1 (Ab M89D3, gift of Dr E. Ferrero) followed by rabbit anti-mouse RPE-conjugated Ab. Cell nuclei were counterstained by Hoechst 33342 (Sigma). For IL-1Ra and vimentin, or CD14, or activated caspase3 co-staining, treatment with goat anti-human IL-1Ra Ab was followed by donkey anti-goat peroxidase-conjugated Ab. Peroxidase activity was revealed by brown staining of oxidized DAB (3,3′-Diaminobenzidine, Dako). Sections were then incubated with anti–vimentin or -active caspase-3 (Chemicon), or -CD14 Ab (Promega Madison, WI), followed by Alkaline Phosphatase labelled polymer (Dako). Alkaline Phosphatase activity was revealed by FAST RED. A rabbit polyclonal anti-IL-1Ra (sc-25444) was used as primary antibody on mouse heart tissue, followed by biotinylated anti-rabbit Ab and streptavidin-peroxidase VIP (purple) staining (Vector Labs, Burlingame, Ca). Mouse cardiomyocyte apoptosis was measured by *in situ* detection of DNA fragmentation (ApopTag, Chemicon). The apoptotic rate (AR) was expressed as the number of apoptotic cardiomyocytes on all cardiomyocytes per field. Cultured cardiomyocytes were stained with goat anti-mouse IL-1Ra and/or IL-1R1 Ab, followed by secondary FITC-conjugated donkey anti-goat Ab, and by either Hoechst 33342 or using the TUNEL fluorescent assay (Roche, Germany). Controls without primary or secondary antibodies were run in all experiments. Observations were carried out by a DM 2000 (Leica, Wetzlar Ge) microscope.

### *In situ* RT-PCR

Expression of IL-1ra RNA in human myocardium samples was evaluated as previously described [20]. IL-1ra specific primers were: 5′-ATGGAAATCTGCAGA GGCCTC-3′; reverse 5′-TGGTTGTTCCTCAGATAGAA GGTCTT-3′. No primer control and no RT control were included in the assay. After the amplification step, slides were counterstained with Vectashield-DAPI (Vector). To demonstrate that the correct target segment was specifically amplified in the in situ PCR reaction, myocardial samples were used in RT in situ PCR overamplification experiments (35 cycles). This procedure allowed the over-production of the IL-1Ra desired amplidicon (140 bp, revealed by agarose gel electrophoresis and ethidium bromide staining) which was detected in the reaction mixture recovered from myocardial samples.

### Semi-quantitative Real Time-PCR

Samples of human or mouse hearts (approximately 50 mg of tissue) or cultured cardiomyocytes (approximately 10^6^ cells) were processed using the GenElute™ Mammalian Total RNA Miniprep Kit, (Sigma-Aldrich, St. Louis, MO). The iScript reverse transcriptase mixture (BioRad Laboratories, Hercules CA) was then used to synthesize the first-strand cDNA, starting from 1 ug RNA as template. Real-time quantitative PCR thermo cycling was conducted using a Rotor-Gene 6000 (Corbett Robotics, Australia). Real-time semi-quantitative amplifications of human IL-1Ra isoforms were conducted by means of Custom TaqMan Gene Expression Assays (Applied Biosystems). Primers and probes were designed for splice variants of the four isoforms of human IL-1Ra, such that Taqman probes spanned the exon-exon junction. TaqMan endogenous controls were eukaryotic 18S rRNA, and human beta actin. A melt curve analysis was performed following every run to ensure a single amplified product for every reaction. Real-time PCR amplifications of murine sIL-1Ra and icIL-1Ra isoforms were conducted using iQ SYBR Green Supermix (BioRad Laboratories) according to the manufacturer’s instructions. Primers were: M-il1rn-s (sense ctcatccttctgtttcattcagag, antisense ccagacttggcacaagacagg, 250 bp), M-il1rn-ic (sense gtttagctcacccatggcttca, antisense ccagacttggcacaagacagg, 251 bp), M-beta actin (sense ggctgtattcccctccatcg, antisense ccagttggtaacaatgccatgt, 154 bp), M-glucuronidase beta (sense ggctggtgacctactggattt, antisense ggcactgggaacctgaagt, 131 bp). Specificity of primers was confirmed by BLAST analysis. Results are expressed as arbitrary mRNA units compared to mRNA expression by normal control mouse heart tissue, or mouse cardiac myocytes cultured in aerobic conditions.

### Coimmunoprecipitation of Caspases with IL-1Ra and Western Blots

Immunoprecipitation was conducted on mouse heart tissue or cultured cardiomyocyte cytosols using polyclonal Abs to IL-1Ra, or caspases, or control IL-1βeta (Santa Cruz), coupled to Sepharose beads plus protein A/G (Santa Cruz). Precipitates were washed in PBS and 100 ug fractions were then boiled in SDS buffer and separated on SDS-PAGE. Blots were probed using monoclonal Abs (Santa Cruz).

### In vitro Caspase Activity

Caspase-3, -6, - 7, and -9 activities were assayed at 22°C, using a Fluor meter plate reader (BMG Labtech Fluostar, Offenburg, Germany). The fluorimetry assays were conducted in the kinetic mode with excitation and emission wavelengths of 400 and 505 nm, respectively. Activity was measured by the release of 7-amino-4-methylcoumarin (AMC) from the synthetic substrate Ac-LEHD-AMC for caspase -9, and Ac-DEVD-AMC for terminal caspases (caspase-3, -7 and -6), respectively. Assay mixtures contained 10^4^ rpm supernatants (50 µg protein) of cell lysates [21], or 50 Units of rh-caspases (BioRad Laboratories), increasing amounts (1–100 µM) of the specific substrate, and caspase buffer [50 mM HEPES, 100 mM NaCl, 1 mM EDTA, 0.1% CHAPS, 10% sucrose and 5 mM dithiothreitol (DTT)]. IL-1β-blocking Abs (R&D System) were used as internal controls. To determine the effect of rhIL-1Ra (Amgen, Thousand Oaks, CA) or rh-xIAP (R& D Systems, Minneapolis, MN) on activity of caspases, assays were performed in the absence or presence of 0.02–2.0 µM IL-1Ra or xIAP. Samples were compared to each other based on the activity of control samples. Data were fitted into the reciprocal Michaelis–Menten equation, and the i_0,5_ values were then derived from the experimental plot, according to Cornish-Bowden [22].

### Statistics

Quantitative results are expressed as mean ± s. e. m., or median and interquartile range for non-parametric variables. SPSS 11.0 for Windows was used for statistical analysis. The ANOVA was used to compare mean between multiple groups with Bonferroni corrected T test used to compare 2 groups at a time. The Mann-Whitney and Wilcoxon tests were used to compare non-paired and paired non-parametric data, respectively. Two-tailed p values <0.05 were considered statistically significant.

## Results

### Synthesis of IL-1Ra in the Human Myocardium

**Figure 1:**
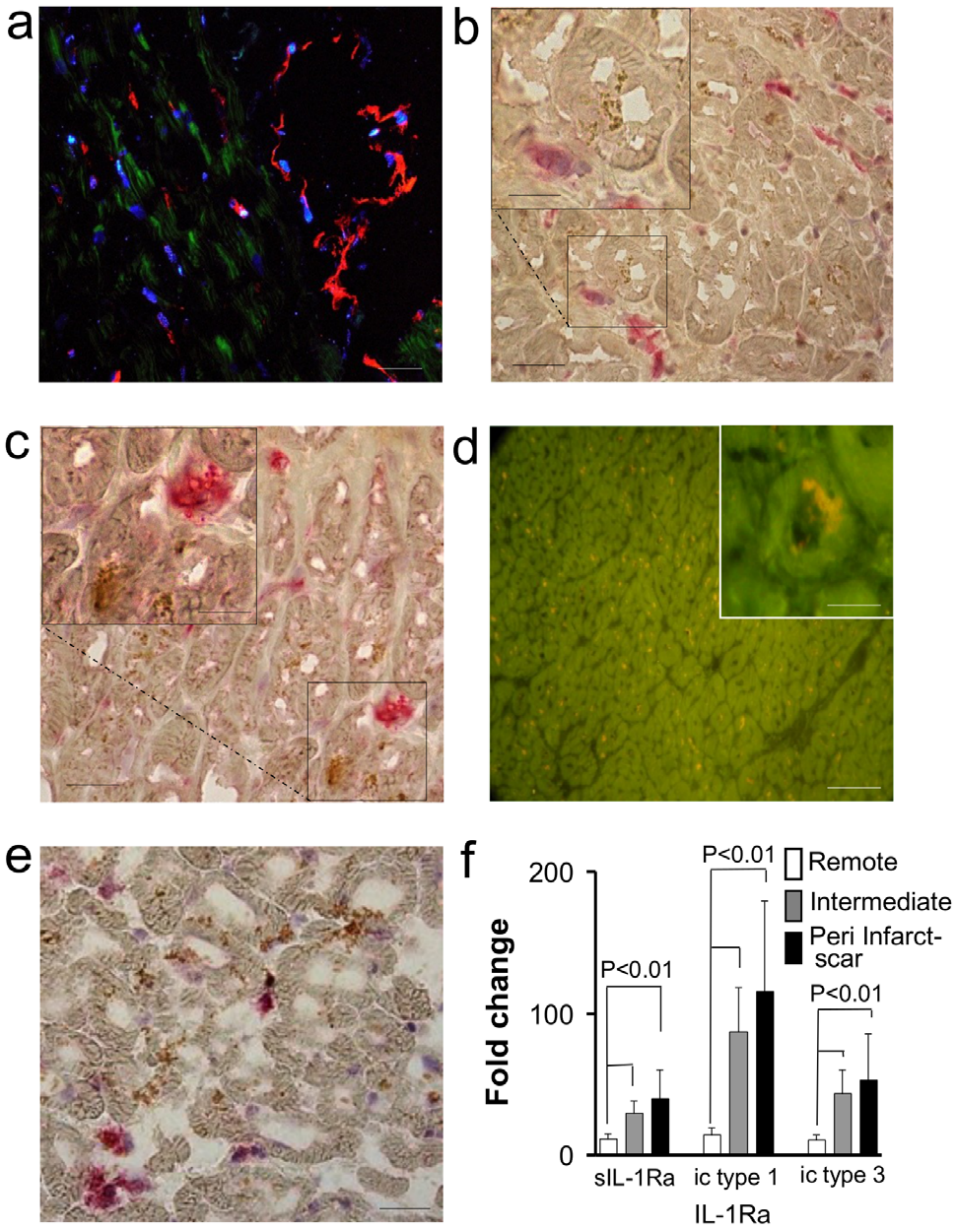
Expression of IL-1Ra in hearts explanted from patients with end stage ischemic heart disease. (a) Immunofluorescence co-staining for IL-1Ra and PECAM-1. Several cardiomyocytes show positive staining for IL-1Ra (green), whereas PECAM-1 positive (red) endothelial cells of myocardial microvessels do not co-stain with IL-1Ra. Cell nuclei are evidenced by DAPI (blue) stain. (b) Co-staining of IL-1Ra (brown) and fibroblast specific vimentin (red), and (c) of IL-1Ra (brown) and leukocyte/macrophage specific CD14 (red). Nuclei are lightly counterstained by Mayer’s Hematoxylin. (d) In situ hybridization for IL-1Ra mRNA. Several cardiomyocytes stained positive for the in situ hybridization in a large area of peri-infarct scar viable myocardium. The inset shows how in situ hybridization is localized mainly in perinuclear areas within cardiomyocytes. (e) Co-staining for IL-1Ra and active caspase3 in a peri-infarct scar area. Besides IL-1Ra positive cardiomyocytes (brown) there are several caspase3-positive cells (red). Bars: a–c, e 20 um, d 40 um, insets 10 um. (f) qRT-PCR analyses of sIL-1Ra and icIL-1Ra (type-1, and type-3) mRNA in ischemic cardiomyopathy, corrected for mRNA expression of β-actin. The graph compares heart regions with macroscopic features of normal blood supply and trophism (remote) to heart areas close to post infarct scars (peri infarct-scar) and regions 1 cm away from the scars (intermediate). The bars show mean ± SE of five experiments.

IL-1Ra expression was investigated in myocardial specimens isolated from heart explants for ischemic cardiomyopathy and multiple previous AMI. In the areas adjacent to old post infarct scars (peri-infarct scar regions), 35% [25–40] cardiomyocytes expressed the IL-1Ra antigen in their cytoplasm. Notably, CD31-positive endothelial cells of myocardial microvessels appeared negative for IL-1Ra antigen. Similarly, vimentin-positive fibroblasts and CD14-positive tissue macrophages evidenced in our samples (less than 1% of the cells, even in proximity of post infarct scars) appeared negative for IL-1Ra immune staining (Fig. 1A–C). The rate of cardiomyocytes expressing IL-1Ra decreased to 18% [5]–[26] in regions 1 cm away from the scars (intermediate regions), and to 2.0% [1.0–5.0] in regions with macroscopic features of normal blood supply and trophism within the same ventricle but several cm away from infarct scars (remote regions: P<0.001 vs. peri-infarct scar regions). RT in situ PCR confirmed the actual synthesis of IL-1Ra in cardiomyocytes (Fig. 1D). The extent of IL-1Ra mRNA and IL-1Ra protein positive cells were virtually identical. A potential link between IL-1Ra expression and cardiomyocyte injury caused by ischemic conditions was then investigated by comparing IL-1Ra staining to apoptosis, as revealed by active-caspase3 [9] and IL-1Ra co-staining (Fig. 1E). Caspase positive cell rates of 2.0% [0.5–2.5] were detected in peri-infarct scar “myocardium at risk” regions [10], vs 0,4% [0.3–0.5] in intermediate and vs 0.17% [0.15–0.19] in remote regions, respectively (P<0.001), thus reflecting the relative proportion of IL-1Ra expression in the same areas. Hearts from subjects virtually free of cardiac disease (controls) showed rates of caspase3 and TUNEL positive cells <0.1% [median 0,04%]. Once established that cardiomyocytes were the prevalent source of IL-1ra, expression of mRNA for IL-1Ra was investigated in human hearts by semi-quantitative real-time PCR. Compared with remote heart regions, the rates of expression of sIL-1Ra and of icIL-1 type 1, and type 3 isoforms were ∼5 fold higher in the regions adjacent to post-infarct scars, and ∼2 fold higher in the intermediate regions (Fig. 1F). Consistent with previous observations in human tissues [23], the mRNA of icIL-1Ra type 2 isoform was not detectable in any of our samples.

### IL-1Ra Synthesis Protects Cardiomyocytes from Ischemia-induced Apoptosis

**Figure 2:**
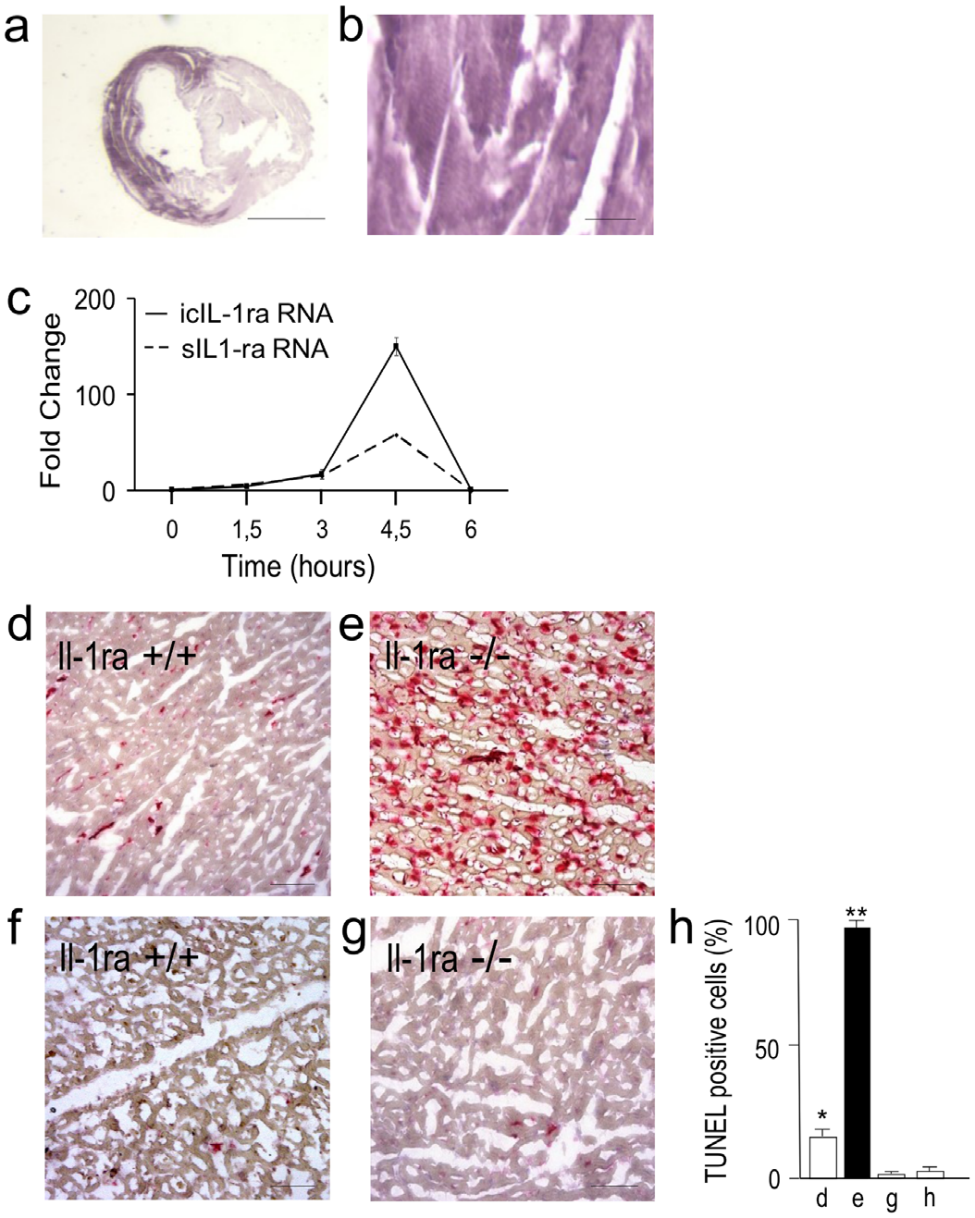
IL-1Ra protects cardiomyocytes from ischemia-induced apoptosis. (a) Hystochemistry of IL-1Ra expression (purple) in the heart following coronary artery ligation in mice: ventricle cross section, and (b) specific, diffuse IL-1Ra staining of cardiomyocytes in the ischemic heart area. (c) Time course of secreted (s) and intracellular (ic) IL-1Ra mRNA expression in the hypoxic heart of WT (Il-1ra+/+) mice. The graphs represent the fold change after normalization with the expression of β-actin. (d) Histology of TUNEL staining (red stain) of Il-1ra+/+ and (e) Il-1ra−/− mouse hearts after 6 hr hypoxia, and of (f) Il-1ra+/+ and (g) Il-1ra−/− mouse hearts not exposed to hypoxia. (h) Rate of TUNEL staining in d-g conditions. Results are means ± SE, n = 3, **p<0.001 for Il-1ra−/− vs control Il-1ra+/+ mouse hearts after 6 hr hypoxia, *p<0.001 for Il-1ra+/+ mouse hearts after 6 hr hypoxia vs hearts not exposed to hypoxia. Bars, a 2 mm, b 20 um; d, e, g, h 40 um.

In order to establish whether ischemia not followed by reperfusion may induce IL-1Ra expression in cardiac myocytes, IL-1Ra synthesis was investigated in mice subjected to an acute myocardial infarction (AMI) protocol by ligation of the proximal left coronary artery for up to 6 hours, an established time limit for survival of myocardial tissue in absence of blood supply, preluding to oncosic-necrosis changes [10]. Histological examination of untreated hearts did not show expression of this cytokine. Treated hearts evidenced a low proportion 5% [4]–[6] of cardiomyocytes expressing IL-1ra in 3 hours-treated mice, whereas strong IL-1ra expression by 95% [92–97] cardiomyocytes (p<0.01, 3 exp.) was evidenced, confined to the ischemic area, in 4.5 and in 6 hours-treated mice (Fig. 2A, 2B), confirming that ischemia was a potential stimulus for IL-1ra cardiomyocyte synthesis. To determine the potential role of IL-1Ra synthesis in protection against ischemia-induced cardiomyocyte death, we used mice lacking IL-1Ra (Il-1ra−/−) [17]. In a wide series coronary ligation experiments performed in previous studies [12] we had experienced that, at histology, the heart area virtually dependent on blood supplied by the ligated artery frequently showed fields of normally perfused tissue, possibly due to collateral vessels. Moreover, 4 to 6 hours after coronary ligation, we evidenced a patchy bordering of the ischemic area by neutrophil leukocytes. Thereafter, to exclude the possible interference with complete ischemia by blood supplied by collateral vessels as well as a virtual contamination by infiltrating leukocytes in the coronary ligation model, we analyzed changes in isolated mouse hearts maintained at 37°C in hypoxic conditions (95%N_2_-5%CO_2_) for various time periods. Quantitative RT-PCR analysis of the hearts from WT (IL-1Ra +/+) mice showed a significant increase in both secreted and intracellular IL-1Ra isoform RNA (Fig. 2C) after 4.5 hr of hypoxia, which decreased to undetectable levels at 6 hr. Notably, at 4.5 hr of hypoxia, the increase of icIL-1Ra RNA was as high as 150.0±5.2 fold, and that of sIL-1Ra RNA was of 58.2±5.4 fold, with respect to control values from hearts immediately after isolation. A low 16% [15]–[17] proportion of TUNEL-positive cardiomyocytes was detectable at 6 hr after heart isolation (Fig. 2D). In contrast, 98% [96–99] TUNEL-positive cardiomyocytes were present in heart samples of mice lacking IL-1Ra at 6 hr (Fig. 2E), indicating that IL-1Ra synthesis actually protects cardiomyocytes from hypoxia-induced death. A very low proportion of TUNEL-positive cardiomyocytes was evidenced in control samples from Il-1ra+/+ (Fig. 2F) or Il-1ra−/− (Fig. 2G) mice not exposed to hypoxia, i.e. 1,1% [0.6–1.4] in Il-1ra+/+ and 2.5% [1.7–3.4] in Il-1ra−/− mice (Fig. 2H).

### The Anti-apoptotic Function of IL-1ra is IL-1R1-independent

**Figure 3:**
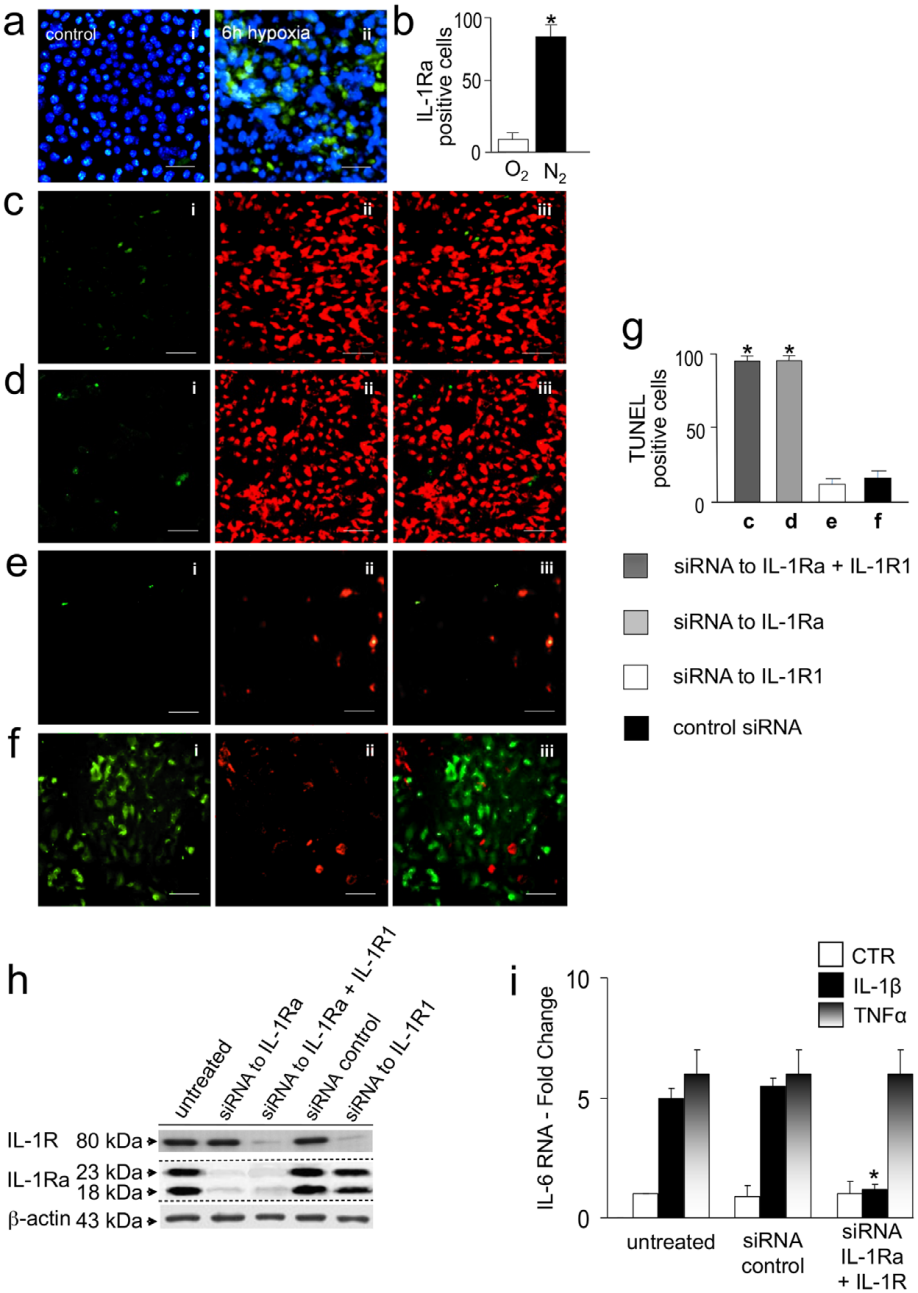
IL-1R1-independent anti-apoptotic function of IL-1Ra. (a) Immunofluorescence of IL-1Ra (green) and nuclear DAPI (blue) staining of cultured mouse cardiomyocytes (HL-1 cells) incubated for 6 hr in normoxia (panel i), or hypoxia (95%N_2_-5%C0_2_ panel ii) conditions, and (b) rate of IL-1Ra positive cells (%) in fig. a conditions. (c) Double-immunofluorescence for IL-1Ra and IL-1R1 (both green, panel i), or (d) for IL-1Ra (green, panel i), or (e) for IL-1R1 (green, panel i), or (f) for IL-1Ra and IL-1R1 (both green, panel i), respectively, together with TUNEL co-staining (red, panel ii) in the same field (merge, panel iii) of cultured cardiomyocytes treated with siRNA to both IL-1Ra and IL-1R1, or to IL-1Ra alone, or to IL-1R1 alone, or with control siRNA, respectively, and exposed to 6 hr hypoxia. Bars, 20 um. (g) Rate of TUNEL positive cells (%) in fig. c-f conditions. Results are means ± SE, and were obtained using three siRNA probes to IL-1Ra. n = 8. *p<0.001 vs controls. (h) Western blot detection of IL-1Ra and IL-1R1 protein expression in fig. c-f conditions. (i) RTqPCR analysis of IL-6 mRNA expression in HL-1 cardiomyocytes treated with siRNA to both IL-1Ra and IL-1R1, or control siRNA, and cultured for 5 hr in the presence or absence of IL-1 beta ((40 pg/ml) or TNF alpha (10 ng/ml), corrected for mRNA expression of beta-actin. The results confirm down regulation of the IL-1 receptor (IL-R1) in siRNA-treated HL-1 cells. The bars show mean ± SE of four experiments; *p<0.001 vs activity of TNF alpha-treated controls.

IL-1Ra expression was further investigated in a cell line of mouse cardiomyocytes (HL-1 cells) [18]. HL-1 cells cultured in hypoxic conditions confirmed that hypoxia is a potent stimulus for IL-1Ra synthesis (Fig. 3A, 3B). RNA interference (siRNA) studies in which synthesis of either IL-1Ra alone, or IL-1Ra together with the IL-1 plasma membrane signaling receptor (IL-1R1) had been down-regulated, confirmed the results obtained in Il-1ra−/− mouse hearts. Cells from these groups subjected to 6hr-hypoxia conditions, showed 96% [94–97] TUNEL-positive nuclei with the double knockdown of IL-1Ra and IL-1R1 (Fig. 3C) as well as with the knockdown of IL-1Ra alone (Fig. 3D) after 6hr hypoxia, vs 14% [13]–[15] TUNEL-positive nuclei in controls treated with siRNA to the IL-1R1 alone (Fig. 3E) or control siRNA(Fig. 3F, 3G). Down regulation of the IL-1R1 in siRNA-treated cells was confirmed in control experiments by Western blot (Fig. 3H). Moreover, RTqPCR analysis of IL-6 expression after stimulation of cardiomyocytes with IL-1β, further confirmed the functional down regulation [3] of the IL-1R1 in siRNA to IL-1Ra and IL-1R1 interfered cells, since siRNA-treated cardiomyocytes increased the expression of IL-6 after stimulation with TNFα, but not after stimulation with IL-1β (Fig. 3I). With respect to cell apoptosis, these studies excluded a potential competitive agonistic activity of IL-1Ra at the IL-1R1 level, since IL-1Ra down-regulated cells were not protected by knocking down of the receptor.

### IL-1Ra Inhibits Mitochondria-activated Caspases

**Figure 4:**
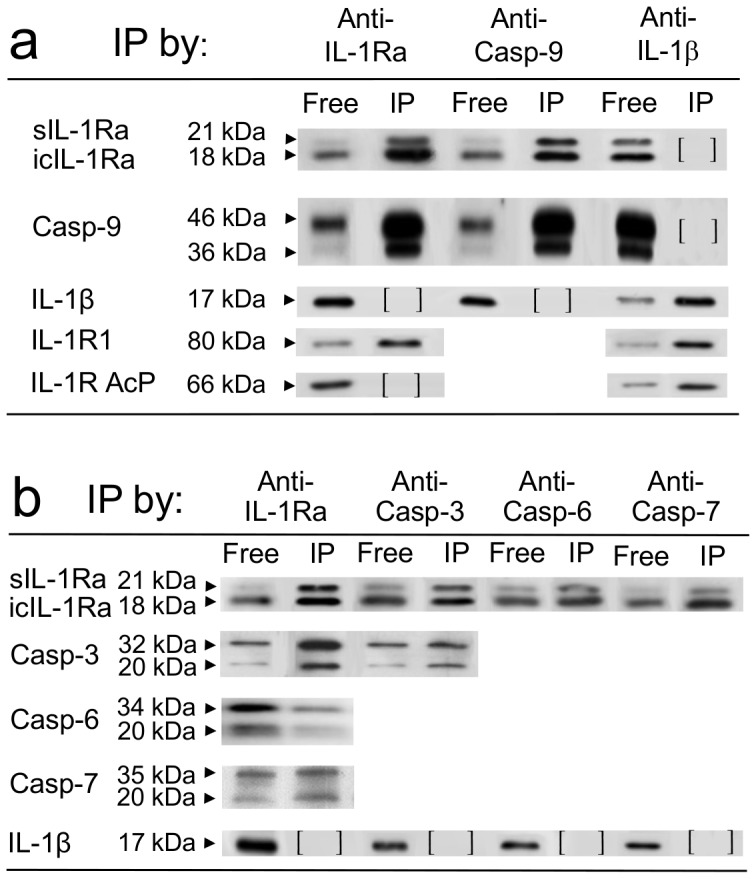
Coimmunoprecipitation of IL-1Ra with mitochondria-activated caspases. (a) Coimmunoprecipitation of IL-1Ra with caspase-9 and (b) with caspase-3, -6, and -7 in cultured HL-1 cardiomyocytes after 6 hr hypoxia. Detection by western blot with monoclonal Abs to caspases or to IL-1Ra, or to control proteins IL-1beta, IL-1 type I receptor (IL-1R1) and IL-1R Ancillary Protein (IL-1R AcP). Proteins immunoprecipitated (IP) by Abs to caspases or to IL-1Ra, or to IL-1beta (control) are compared to unbound (free) supernatant proteins. The data are compiled from different gels in three separate experiments; [ ] not detected.

**Figure 5:**
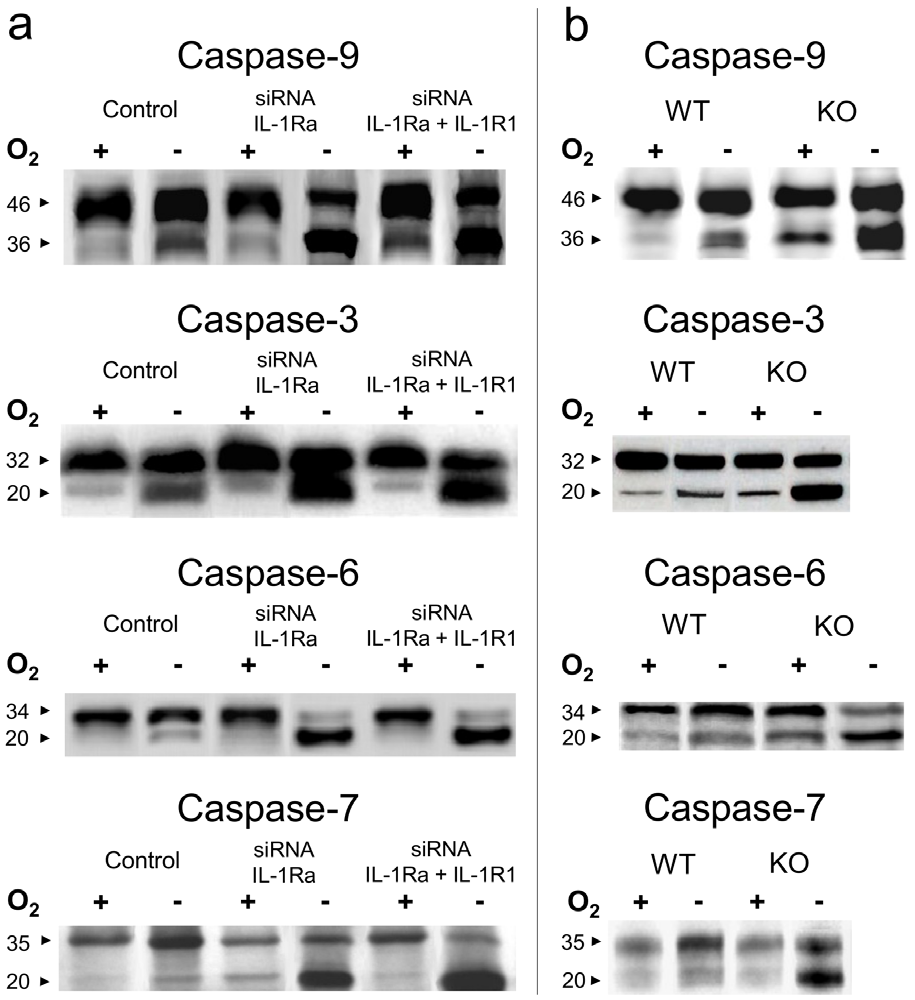
Cleavage of mitochondria-activated caspases in cardiomyocytes lacking IL-1Ra. (a) Western blot of caspase-9, -3, -6, and -7 from cultured HL-1 cardiomyocytes untreated (controls) or treated with siRNA to IL-1Ra RNA alone or both IL-1Ra and IL-1R1 RNAs, and then incubated for 6 hr in normoxia (O_2_+) or hypoxia (O_2_−) conditions. (b) Western blot of caspase-9, -3, -6, and -7 from Il-1ra+/+ (WT) or Il-1ra−/− (KO) mouse hearts, before (O_2_+) and after (O_2_−) 6 hr hypoxia. 3 exp.

**Figure 6:**
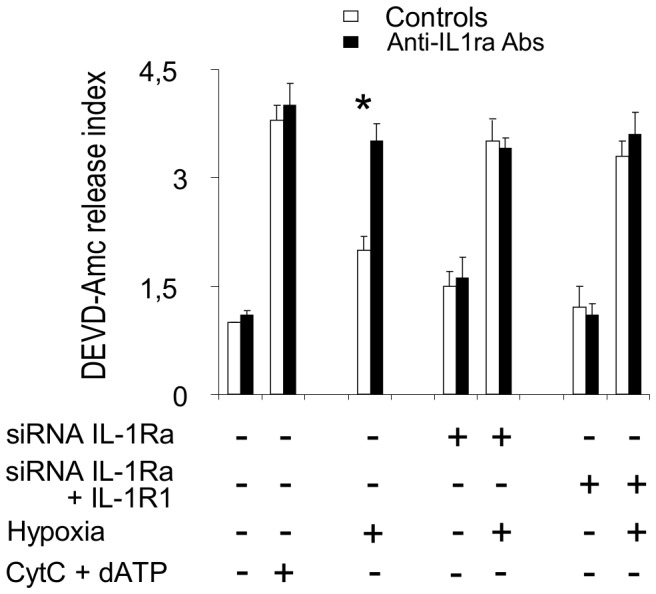
In vitro activity of terminal caspases in cardiomyocytes lacking IL-1Ra. Activity of mitochondria-activated terminal caspases in cytosols of cultured HL-1 cardiomyocytes untreated or treated with siRNA to IL-1Ra RNA, or both IL-1Ra and IL-1R1 RNAs, and then incubated for 6 hr in normoxia or hypoxia conditions. Ac-DEVD-AMC assays compare enzyme activity in the absence (controls) or presence of anti-IL-1Ra Abs. Bars show means ± SE of 3 exp.; *p<0.01 vs activity of controls.

**Figure 7:**
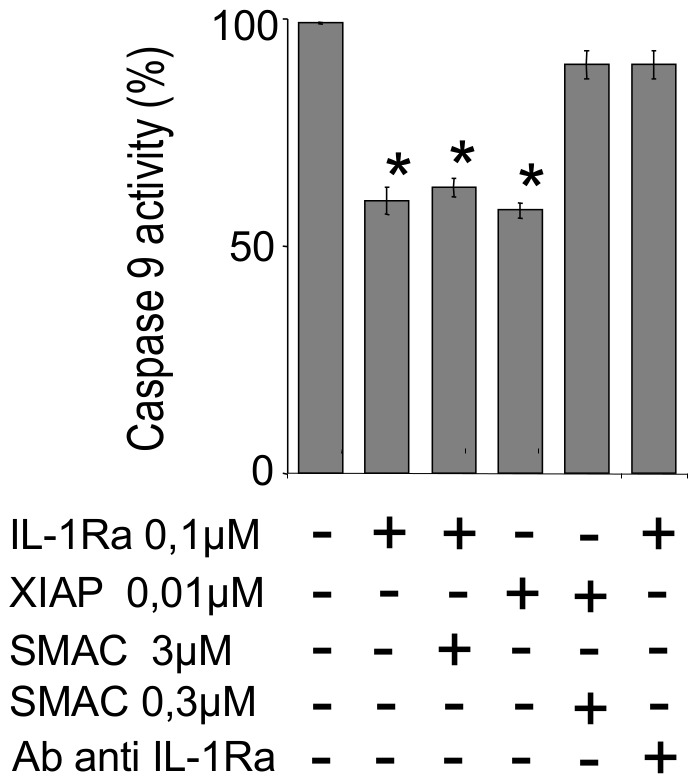
IL-1Ra is not inhibited by SMAC. Caspase-9 inhibition by IL-1Ra or Xiap in the presence and absence of the Xiap inhibitor SMAC, or Ab to IL-1Ra. Bars show means ± SE of 3 exp.; *p<0.01 vs activity of controls.

We then sought to elucidate the intracellular mechanism by which IL-1Ra would inhibit cell apoptosis. Since activation of caspase-9 by release of cytochrome-C from mitochondria plays a central role in ischemia-induced apoptosis [24], [25], we looked at the potential interactions of IL-1Ra with caspase-9 and with caspases -3, -6, and -7, acting downstream caspase-9 activation in this cell death pathway. The co-immunoprecipitation experiments using anti-IL-1Ra or anti-caspase Abs coupled to sepharose beads demonstrated interaction of both intracellular [4] and secreted IL-1Ra isoforms with caspase-9 (Fig. 4A) and with caspase-3 (Fig. 4B), whereas co-immunoprecipitation of IL-1Ra with caspases -6 and -7, appeared more limited (Fig. 4B). No interaction with caspases was evidenced for IL-1β and no interaction with IL-1Ra was evidenced for IL-1R1 ancillary protein (IL-1R AcP), respectively, used as internal controls in these experiments. Resting caspases exhibit relative molecular weights (Mw) different from their activated, cleaved fractions. We compared Mw of caspases in HL-1 cells cultured in normoxic and 6hr-hypoxia conditions, and with cell preparations pre-treated with siRNA to IL-1Ra or to IL-1Ra and IL-1R1 (Fig. 5A). The Mw of protein recognized by anti-caspase-9 Abs from cells cultured in normoxic conditions was 46 kDa, which corresponded to the Mw of resting-caspase, with a smaller proportion of 36 kDa caspase-9, compatible with cleavage during the extraction procedure. After 6hr hypoxia, a small proportion of caspase-9 from untreated cells was detected again at 36 kDa, suggesting limited activation of caspase-9. In contrast, in siRNA-treated cells the bulk of caspase-9 appeared at 36 kDa Mw, indicating increased activation of this enzyme in IL-1Ra deficient cells with or without concomitant downregulation of IL-1R1. Similar results were obtained by studying caspase-3, -6, and -7, as well by comparing caspase-9, -3, -6, and -7 Mw (Fig. 5B) in IL-1Ra +/+ (WT) and mutant Il-1ra−/− (KO) 6 hr hypoxia-treated or untreated mouse hearts. As an additional control, the effect by siRNA inhibition of IL-1Ra, or both IL-1Ra and IL-1R1 synthesis on mitochondria-dependent caspase activation in cultured cells was confirmed by incubating cytosols from siRNA-treated, or untreated-control cells, in the presence of the caspase-3, -6, and -7 common fluorigenic peptide substrate Ac-DEVD-AMC, and measuring residual enzyme activity by spectrofluorimetry. Limited caspase activity was measured in cytosols from normoxic controls unless cytochrome c and dATP were added to cytosols. The activity in controls incubated for 6 hr in hypoxic conditions was significantly enhanced by addition of anti-IL-1Ra Abs (p<0.001), whereas activity of siRNA-treated cells cultured in hypoxic conditions peaked without addition of Abs to IL-1Ra. These results confirm IL-1Ra inhibition of mitochondria-dependent caspase activation in control cells, absent in siRNA interfered cells (Fig. 6). To compare the inhibitory effect by IL-1Ra on each of mitochondria-activated caspases, activity of rh-caspases was measured by spectrofluorimetry in the presence or absence of rhIL-1Ra. In our conditions, we obtained Km values of 85 µM for caspase-9, and of 5.3 µM, 71 µM and 21 µM for caspase-3, -6 and -7, respectively, which were in accordance with previously published data [26]. At substrate Km-concentrations, IL-1Ra inhibited caspase-9 with *i*_0,5_ values of 0.31 µM. Caspase-3, -6, and -7 were also inhibited by IL-1Ra, but at concentrations considered biologically unlikely [27], since *i*_0,5_ for rhIL-1Ra inhibition of caspase-3, -6, and -7, were 2.5 uM, 2.2 uM, and 1.3 uM, respectively (4 exp., p<0.01), thus suggesting that IL-1Ra inhibition of caspase-9 in intact cells is, most likely, indirectly responsible for down regulation of downstream caspases. Smac/diablo is a protein that is highly expressed in the heart [28], [29]. It is released from the mitochondria along with cytochrome c upon induction of apoptosis. By binding and sequestering naturally occurring caspase inhibitors (IAPs), Smac disinhibits caspase activation. In our assays, the IAP family member Xiap (50 nM) potently inhibited caspase-9, as expected from previously reported results [30]. To compare the effect of Smac on caspase inhibition by Xiap and by IL-1Ra, rh-caspase-9 activity was measured in the presence of either rh-IL-1Ra or rhXiap, and in the presence or absence of rhSmac (Fig. 7). In these experiments, Caspase-9 was equally inhibited by 50 nM Xiap (44±2% inhibition) or by 100 nM IL-1Ra (41±3% inhibition). As expected, however, Xiap inhibition was abolished in the presence of Smac (1–1.5 µM) whereas IL-1Ra inhibition was not influenced at all by up to 3 µM concentrations of Smac, i.e. 30 fold higher than IL-1Ra concentration. Notably, IL-1Ra inhibition of Caspase-9 activity was abolished in the presence of anti-IL-1Ra Abs, used as internal control in our assays.

## Discussion

This study shows synthesis of secreted and intracellular IL-1Ra isoforms (sIL-1Ra, icIL-1Ra) by cardiomyocytes in ischemic conditions. IL-1Ra synthesis was observed in cardiomyocytes, and not in the inflammatory cells or cells of coronary vessels, in patients with severe ischemic myocardial disease, particularly in areas of the heart with increased apoptosis, as well as in mouse cardiomyocytes exposed to enduring ischemic conditions. Previous studies have shown a very early increase of IL-1Ra, which preceded the appearance of markers of necrosis in the serum of patients with AMI, particularly if the AMI was anticipated by pre-infarction angina [6], [7], suggesting that a condition preceding necrosis may have caused IL-1Ra production. In the present study, we observed IL-1Ra synthesis in the areas of old post infarct scars in the human myocardium. Moreover, within scar areas, we noticed a generalized although not uniform increase of IL-1Ra positive cells, as compared with other areas of the ventricle, paralleled by increased rates of myocardiocyte apoptosis. Taken together, these results suggest that ischemia triggers IL-1Ra synthesis in cardiomyocytes. It is conceivable that the elevated serum levels of IL-1Ra in coronary patients are actually due to IL-1Ra released by ischemic cardiomyocytes, and may have potential clinical interest for the early diagnosis and prognosis of myocardial disease.

**Figure 8:**
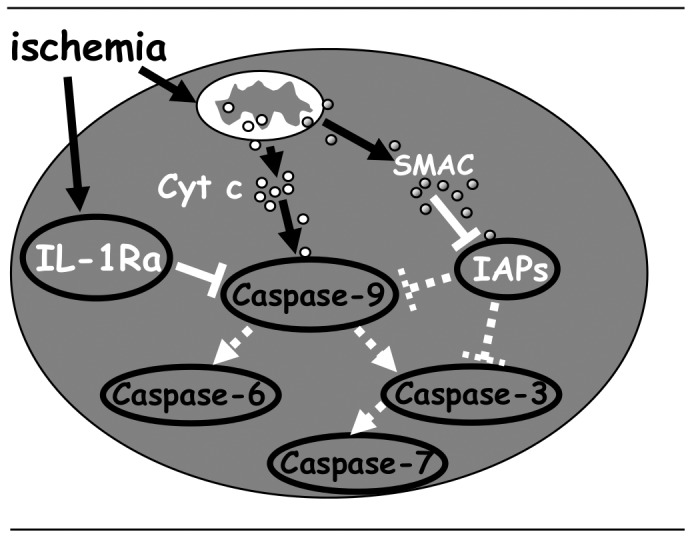
Hypothetical model of IL-1Ra in the inhibition of apoptosis. Smac released from ischemia-induced mitochondria which inhibits the neutralizing effect of IAPs on caspases. Ischemia-induced IL1ra interacts with caspase-9 and blocks cell death.

The results also indicate that IL-1Ra potently inhibits hypoxia-induced apoptosis in mouse cardiomyocytes, primarily by interfering with caspase-9 activity. Further studies are needed to quantify the actual role played by each IL-1Ra isoform (icIL-1Ra, sIL-1Ra) in mediating this intracellular function of IL-1Ra. However, the binding of both isoforms to caspases evidenced in supernatants of ischemic cells (Fig. 4) may suggest a combined activity of sIL-1Ra and of icIL-1Ra in this function. The activation of caspase-9 plays a critical role in ischemia-induced apoptosis [31], [32]. In response to hypoxia, the mitochondria release cytochrome c into the cytosol which associates with Apaf-1 and ATP, triggering activation of caspase-9. Committed caspase-9 further activates downstream effector caspases -3, -6, and -7 that account for cell phenotype changes associated with apoptotic cell death [31]. Release of cytochrome c in cultured cardiomyocytes has been reported in models of hypoxia [33]. Moreover, cytochrome c release from mitochondria and caspase-3 activation were observed in heart samples from patients with end stage cardiomyopathy [24]. In response to biomechanical and mild ischemic stress, substantial cardioprotection is achieved by Apoptosis Repressor with Caspase recruitment domain (ARC) [34], an anti apoptotic factor that prevents cytochrome c release and subsequent cell death, by interfering with Bax activation. However, exposure of cardiomyocytes to ischemia, hypoxia, or oxidative stress leads cytochrome c release as well as rapid down regulation of ARC protein levels, thereby abolishing the cardioprotective role of ARC [34]. In the present study, we show that ischemia stimulates up to 160 fold increase per time unit of IL-1Ra production, and that IL-1Ra specifically binds, and inhibits caspase-9 activity, with *i*_0,5_ in the nano molar range, and activity of caspases 3, -6 and -7 with *i*_0,5_ in the micro molar range. These results suggest that IL-1 may substitute for ARC in enduring ischemic conditions. Among the regulators of apoptosis, considerable interest has been focusing on the inhibitors of apoptosis protein (IAP) family, which is an evolutionary conserved family of proteins that prevent cell death across species. X-linked IAP (Xiap), a currently known human member of the IAP family, was reported to inhibit caspase-9 at 10-9 M concentrations [35]. Other IAP family members IAP-1 and -2, and NAIP were reported to inhibit caspase-3 and -7 at 10^−8^–10^−7^ M concentrations, but not to inhibit caspase-9. The same applies to survivin, a IAP expressed in human embryonal tissues and tumor lines but not in adult tissues. Survivin inhibits caspases 3 and 7 as potently as Xiap, with *K*is of 10^−10^ M [36]. Our data indicate that IL-1Ra inhibits caspase-9 with *i*_0,5_ of 10^−7^ M, representing 2 logs lower potency than Xiap, but comparable to the inhibitory effect with respect to caspase-3 and -7 reported for IAP-1 and -2 [36]. Thus while the *i*_0,5_ obtained for IL-1Ra reflects structural differences between IL-1Ra and the IAP family members that affect how well they bind to and inhibit specific caspases, presumably it is necessary for this protein to be present in the cell at higher concentrations than XIAP to achieve the same level of protection against caspase-9, -3, -6 and -7. The observed ∼150 fold increase of IL-1Ra RNA production by ischemic cardiomyocytes may account for its potential role as a physiologically relevant inhibitor of caspase-9 and, possibly, also of caspase-3, -6 and -7 mediated apoptosis in ischemic conditions. Smac, a mitochondria protein released into the cytosol in response to some of the apoptotic stimuli, including ischemia [28], [29], was found to promote caspase activation by binding and neutralizing the IAPs, including XIAP, IAP-1, and IAP-2. Notably, in our assays the anti-caspase-9 activity of IL-1Ra was not affected at all by up to 30 fold higher concentrations of Smac, suggesting that IL-1 may substitute for IAPS to inhibit mitochondria activated caspases in ischemic conditions (Fig. 8).

Recently, Larsen et al. (2009) [37] reported that treatment with recombinant IL-1Ra caused a significant and long lasting improvement of β-cell function in type II diabetic patients bearing a IL-1Ra gene polymorphism associated to reduced IL-1Ra β-cell expression. Moreover, Aksentijevich el al. (2009) [38] and Reddy et al. (2009) [39] reported an hitherto unknown association of a severe autoinflammatory syndrome in 10 patients with homozygous mutations of IL1RN, the gene encoding IL-1Ra, leading to the definition of a new syndrome, deficiency of the Interleukin-1 receptor antagonist [DIRA] [3]. Whether the pathogenetic component of β-cell impairment cured with exogenous IL-1Ra and the pathogenesis of the DIRA syndrome are to be reconducted to the failure of extracellular IL-1Ra to compete with IL-1 at the receptor level and, hence, unopposed proinflammatory IL-1 signaling, or for concomitant lack of intracellular IL-1Ra function remains unclear. It seems possible, however, that the lack or partial alteration of IL-1Ra anti-apoptotic function may account, at least in part, for cell loss in ischemic diseases in which impairment of mitochondria is responsible for the induction of cell apoptosis.
